# Light Pollution in Ultraviolet and Visible Spectrum: Effect on Different Visual Perceptions

**DOI:** 10.1371/journal.pone.0056563

**Published:** 2013-02-18

**Authors:** Héctor Antonio Solano Lamphar, Miroslav Kocifaj

**Affiliations:** ICA, Slovak Academy of Sciences, Bratislava, Slovak Republic; University of Tennessee, United States of America

## Abstract

In general terms, lighting research has been focused in the development of artificial light with the purpose of saving energy and having more durable lamps. However, the consequences that artificial night lighting could bring to the human being and living organisms have become an important issue recently. Light pollution represents a significant problem to both the environment and human health causing a disruption of biological rhythms related not only to the visible spectrum, but also to other parts of the electromagnetic spectrum. Since the lamps emit across a wide range of the electromagnetic spectrum, all photobiological species may be exposed to another type of light pollution. By comparing five different lamps, the present study attempts to evaluate UV radiative fluxes relative to what humans and two species of insects perceive as sky glow level. We have analyzed three atmospheric situations: clear sky, overcast sky and evolving precipitable water content. One important finding suggests that when a constant illuminance of urban spaces has to be guaranteed the sky glow from the low pressure sodium lamps has the most significant effect to the visual perception of the insects tested. But having the fixed number of luminaires the situation changes and the low pressure sodium lamp would be the best choice for all three species. The sky glow effects can be interpreted correctly only if the lamp types and the required amount of scotopic luxes at the ground are taken into account simultaneously. If these two factors are combined properly, then the ecological consequences of sky glow can be partly reduced. The results of this research may be equally useful for lighting engineers, architects, biologists and researchers who are studying the effects of sky glow on humans and biodiversity.

## Introduction

Continuously growing night lighting is recognized as a serious ecological problem of the last decades. Light pollution (LP) represents a negative impact on exposed living organisms, including human beings, and usually is the effect of an unnecessary light that results in a high amount of wasted energy. LP is not as widely known as it should be, but it is increasingly studied to the extent that many countries and organizations are implementing regulations and recommendations for its treatment.

The problem has an unfavorable influence on the life conditions of biological species, including humans. Unnecessary artificial light disturbs the natural night cycles and makes serious ecological imbalances to the life of the planet. All these modifications in the biodiversity can produce serious problems, such as ecological changes of some important species [1]. The earlier studies on the LP effect in animals were performed with the intention to identify the artificial night lighting issues in different species, mainly amphibious, lepidopterans, fishes and birds [2]–[8]. That was the beginning of an extended research labor with diverse investigations showing the negative impacts of LP and sky glow over a variety of biological species, not only animals but also the flora [1], [9]. In animals, LP produces a physiological and behavioral alteration that could be the result on changes in reproduction, predation and orientation habits. It is a fact that LP creates disorientation in birds during their migration flights, they crash into artificial constructions possibly resulting in death from physical damage, starvation, dehydration and predation, among other problems [10], [11]. Assuming that the migratory birds use the stars for orientation in their nocturnal navigation, sky glow could wash out the natural brightness of the star and then produce also disorientation in these birds. Some species of salmon experience changes on melatonin from certain artificial light sources [12], [13], and more than a few bat species could be damaged if they emerged during the day; this is the reason why they do not have ultraviolet (UV) filters in their eyes. These kinds of bats are nocturnal and have a reduced tolerance to lighting in visible (VIS) spectrum but have tolerance of red light and infrared light better than white light [14]. E.g. the nectar bat Glossophaga soricina is sensitive to UV range of the spectrum [15].

The insects are one of the classes of living creatures influenced by artificial light [16], [17], many of the lepidopteran species are attracted by artificial light with some variations between species [18]–[21]. LP could be the factor of their population level changes in Great Britain [22], where some researchers also found a notable biodiversity loss of moths [23]. The wavelength range related to the visual ecology of insects (including fly insects) covers UV spectrum [24]. There are certain variances among individuals and populations; however, all those differences are located between 340 and 400 nanometers [25]. This might be the reason for the increase of bat predation on moths, since LP in UV spectrum may affect the ability of moths to perceive the ultrasonic sound used by bats to locate prey [26]. For instance, the range of the spectrum to which the frontal eye of A. Macaronius is sensitive is located from 300 to 480 nm. The receptor types in some species of butterflies, dragonflies, and Hymenoptera cover visual ranges from 300 to 700 nm. It is possible to infer that insects are mainly UV sensitive and that part of the spectrum may produce oxidative stress [27].

The artificial night lighting has also adverse impacts on the human physiology. The main influence of sky glow and artificial light in humans could be the alteration of the circadian function [28], [29]. The exposure to artificial night lighting produces a dysfunction in the melatonin levels in humans and in their circadian pacemaker [30]. These variations can have a negative effect to the health of shift workers [31]. As well, the alterations in the pineal gland function due to exposure to high levels of artificial light during the night cause a suppression of pineal melatonin production in humans [32], [33]. In a minor manner, LP and sky glow may contribute to disorders including depression, insomnia, among others [34].

An evaluation of sky glow levels under various meteorological conditions is a complex task that can be realized numerically. In the present work, a two-stream approximation is used to compute the downward radiative fluxes in both UV and VIS spectral bands. The two-stream concept considers a horizontally homogeneous atmosphere that is subdivided to a set of plane-parallel layers with well-defined physical properties. Basically, the concentrations of atmospheric constituents (such as air molecules, aerosol particles, water vapor, etc) can differ from layer to layer. The aerosols are one of the most unstable components showing a large effect on the optical properties of the atmosphere. The size and shape distributions, as well as the material compositions of aerosol particles can be altered greatly with altitude. Therefore, the optical properties of atmospheric layers differ significantly depending on the atmospheric model. With the two-stream approximation, the downward and upward radiative fluxes can be computed subject to boundary conditions. In our case, the downward radiance at the top of the atmosphere is considered to be zero, while the upward radiance at the ground is determined as a sum of direct uplight and reflected light. The direct uplight represents a fraction of light that is emitted directly upward. The reflected light comprises (diffuse) reflection from various surfaces, including buildings, trees, roadways, etc. The light beams propagated upwards are scattered and attenuated everywhere in the atmosphere. This process can be quantified when solving the radiative transfer equation for selected spectral bands. For more details consult the study [43] and the papers cited therein.

In this paper we simulate the downward radiative fluxes as perceived by humans under scotopic vision conditions [53]. Also, the corresponding effects are evaluated for two different species of insects. Graphics are made to show a comparative analysis of radiative fluxes that can be detected in both UV and VIS spectral bands and consequently used in the determination of the basic features of sky glow affecting humans and insects. The radiance data in W⋅m^−2^⋅sr^−1^⋅nm^−1^ from five different lamps were analyzed considering only their emission spectra from 350 nm to 780 nm. The lamps taken into consideration are representative samples for what is usually used in public lighting.

## Materials and Methods

### 1. The species

**Figure 1:**
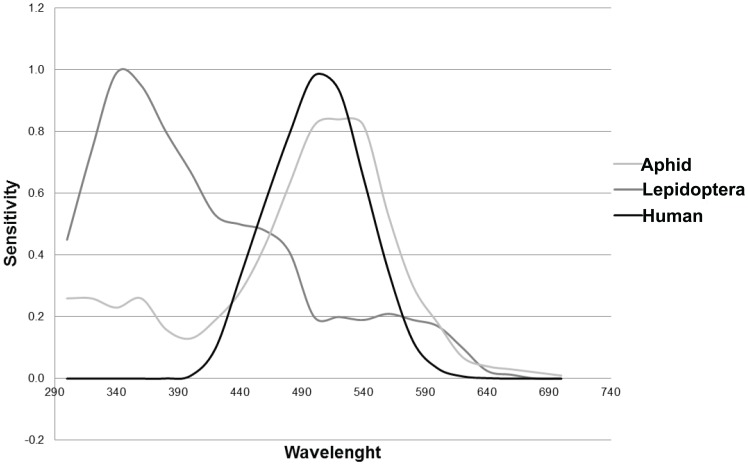
The spectral sensitivity of the dark-adapted eye of alate M. persicae (an aphid species), the spectral sensitivity of Narathura bazalus (a lepidoptera species) and the scotopic spectral sensitivity of humans are considered here.

In our numerical experiment we took into account the scotopic human sensitivity and the sensitivity of two different insect species: lepidoptera narathura bazalus and aphid alate m. persicae (dark adapted eye). The average spectral sensitivities of their visual perceptions of brightness are depicted in the Fig. 1. [35], [36]. The narathura bazalus (butterfly) is diurnal; however, previous studies have reported that diurnal lepidopteran species are attracted to artificial light sources during the night [37], [38]. This butterfly is showing a wide sensitivity to the UV spectrum, therefore it is of great motivation to determine the quantity of downward UV radiative flux perceived by this insect. The alate m. persicae is also diurnal, but a nocturnal behavior with attraction to streetlamps has been demonstrated for this insect [17]. For humans, the photopic vision is still active if the individuals are being exposed to street lighting (e.g. in downtown). However, the photopic vision can change to mesopic vision when a hypothetical observer is moved from a city to a darker place such as e.g. natural space. If the distances to the light sources are large enough (or if the total emission powers and/or the number of lamps are both sufficiently low) a transition from mesopic to scotopic vision could takes place. It is therefore uncertain what kind of sensitivity has to be considered in various situations. There is no doubt that the effects of different lamp emissions on the sky glow can be evaluated reasonably after implementing a common normalization, i.e. a generalization of the computational results would be easily possible if both the sky glow and emissions are evaluated against the same base. In this paper we analyze the sky glow effects relative to the human scotopic vision.

Due to the availability of spectral sensitivity curves sky glow can be studied for all of three organisms discussed above. Although an extensive research has been carried out on the effects of direct light sources to these species, no one study satisfactorily documents the corresponding consequences of sky glow. However, sky glow is a problem that deserves a more detail examination since these species show nocturnal habits. The three species discussed in our paper were chosen carefully with the purpose to identify sky glow effects for living organisms with discrepancies in their spectral sensitivities; the aphid night vision might present similarities with the human scotopic vision, while the spectral sensitivities of lepidoptera and humans are completely different (see Fig. 1). The detail analysis of such discrepancies is a great motivation for giving this targeted study.

### 2. The light sources

Five lamp types, traditionally used for artificial night illumination, were considered in this paper. [39], [40], [41].

**Figure 2:**
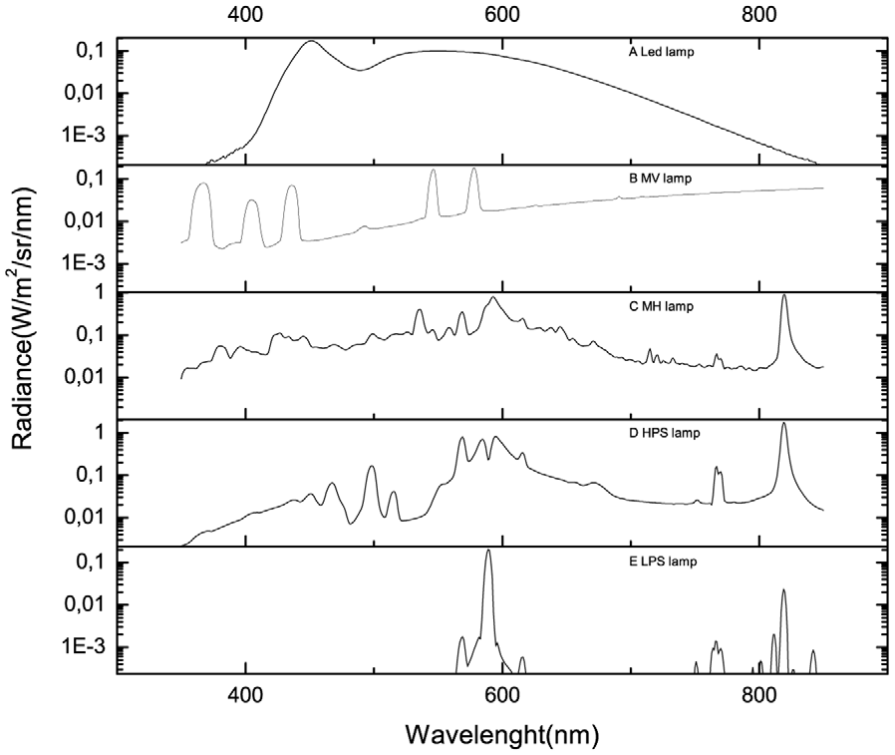
The lamps are the main component of artificial lighting systems designed for public purposes. For this reason these are considered as sky glow sources. The data for the graphs was obtained from The US Dept. of Commerce/NOAA/NESDIS/NGDC website. [39], [40], [41].

- Light emitting diodes (LED), see Fig. 2-A. LED lamps are used in many devices including public lighting. However, such a usage of LED lamps is intensively discussed by environmental researchers who suppose these light sources could have adverse effects on humans and animals by inhibition of melatonin, mainly for the exposure related to blue light [41].
- Mercury vapor (MV) lamps with dominant UV emission spectra. It is possible that these could be the least suitable to be included in lighting installations near the natural parks (see the spectral radiance in Fig. 2-B).
- Metal halide (MH) lamps. MH lamps have a daylight colour rendering index producing an appearance as daylight (see the spectrum in Fig. 2-C). Due to this fact, these lamps are used not only for public lighting but also for filming production and advertising applications.
- High pressure sodium (HPS) lamps with emissions over a comprehensive part of the spectrum (see Fig. 2-D). The light color of these lamps varies between the golden and orange color.
- Low pressure sodium (LPS) lamp (see Fig. 2-E). The emissions of this lamp are limited to a small part of the VIS spectrum. For this reason these lamps could have a quite low attraction to insects.

The HPS-normalized emissions of the five lamps used in our research are summarized in the Table 1. The HPS lamp was taken as a reference light source since it is very frequently used for public lighting purposes in Europe. A more detail description of the normalization concept can be found in the first paragraph of Sec. 3.2.

**Table 1:** Characteristics and relative emissions of the five light sources.

	LED	HPS	LPS	Mercury Vapor (MV)	Metal Halide (MH)
Characteristics					
CCT	6273	2056	1807	4100	3610
CRI	100	17	0	40	65
Lumens	3604	16000	1570	3000	9000
Watts	54	170	18	75	100
Relative emissions					

	LED/HPS		LPS/HPS	MV/HPS	MH/HPS
Radiative (VIS) (380–780 nm)	0,51	1	0,05	0.27	0.31
Radiative (UV) (350–400 nm)	0,05	1	0,00	3,85	2,50
Luminous	1,27	1	0,02	0,19	0.40
Alate m. persicae (350–700 nm)	0,83	1	0,05	0,19	0,33
Narathura bazalus (350–700 nm)	0,76	1	0.06	0,34	0,42

The Tab. 1 can be read in different ways. For instance, if a constant radiative emission is required independent of lamp-type, then two LED lamps are needed to supply the VIS emissions by one HPS lamp. However, if the human perception is considered, one can easily recognize that LED lamp emits about 27% more than HPS lamp. In this case only 4 LED lamps are necessary to produce the luminous energy equivalent to 5 HPS lamps. Note that all these evaluations were made for direct emissions of the lamps considered and not for a diffuse radiation of a night-sky that can be detected at the ground as a diffuse irradiance 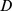.

### 3. The numerical experiment

Two-stream concept is designed to determine both the downward and upward radiative fluxes irrespective of geometry and lamp output patterns. We have implemented Eddington's (two-stream) approximation that is well applicable for the light sources distributed randomly over a large territory, i.e. for the illumination systems with kind of spatial homogeneity from macroscopic point of view. Eddington's approximation dictates that the intensity at any altitude behaves like 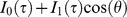, where 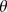 is the zenith angle, 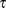 is the atmospheric optical thickness and 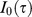 and 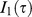 are specific intensity functions (consult e.g. [52]). Note that there is a direct relation between the optical thickness and the altitude level. Accepting the boundary conditions (such as a reflective surface at the bottom interface of the atmosphere and a non-reflective top of the atmosphere), the intensity function at the ground will read as 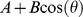. Here 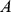 and 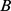 are constants satisfying the requirement that integral from 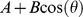 over the upper hemisphere equals to the total luminous flux emitted upward. Since the problem is solved for quasi-homogeneous distribution of the ground-based light sources, a directionality of bulk emissions from a large complex of radiating sources is efficiently averaged. As a consequence, the mean emission function becomes symmetric along vertical axis (i.e. azimuthal variability of intensity-field will either disappear or be at least strongly suppressed). While we performed the comparative computations with HPS as a reference light source, we only need to specify the emissions from this type of light source. The emissions for other types of light sources are computed relatively to HPS. For HPS we required to have the diffuse illuminance of 30 mlx for standard cloudless atmosphere with aerosol optical thickness of 0.5.

The electromagnetic radiation that is emitted by ground-based light sources or reflected by the ground propagates through the atmosphere and undergoes scattering and absorption processes. Part of the electromagnetic energy can be scattered and directed toward the ground, and consequently recorded by a measuring device. Large sets of commercial radiometers are available to detect diffuse irradiance 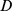 at a horizontal surface. In order to interpret these measurements correctly, the principles of radiative transfer in the atmospheric environment have to be considered. The diffuse irradiance can be expressed as a function of the spectral radiance 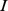. Note that spectral radiance of a sky element 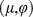 characterizes the radiant energy propagated within a solid angle 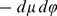 and has dimension W⋅m^−2^⋅nm^−1^⋅sr^−1^. The 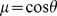 is cosine of the zenith angle 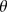, and 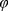 is the azimuth angle. If 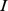 is known, the diffuse irradiance (W⋅m^−2^) is computed as follows
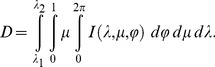
(1)


The integration in Eq. (1) runs over the spectral band bounded by wavelengths 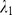 and 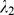. It is evident that detectors embedded into different radiometers may show different spectral responses 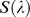, so it is convenient to write Eq. (1) in a more general form:
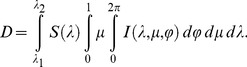
(2)


Using a specific filter with spectral transmittance 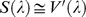, the perception of the human visual system can be simulated. Here 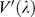 is a function of the scotopic vision [41], [42]. For this research, we have computed the irradiances in UV and VIS spectral bands based on Eq. (1). In accordance with this methodology and applying the Eq. (2) under distinct atmospheric conditions [43], we will evaluate the visual perception of sky glow by different species. Specifically, the aerosol and cloud optical thicknesses as well as the amount of precipitable water are calculated over predefined ranges. The diffuse irradiances 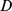 are computed consequently. The vertical stratifications of atmospheric constituents (such as air molecules, aerosols, precipitable water, ozone and other gases) are kept fixed. We have used the two-stream approximation to evaluate sky glow originated from the specific lamp-types. In general, light-pollution problem is dependent upon geometry, especially for sparsely distributed light sources in the vicinity of a measuring point. However, the computations accepting a very detail topology of an ambient environment (comprising illumination system, obstacles, terrain, etc.) become CPU and MEM intensive and thus unattractive for more general analysis. In addition, the results obtained this way would be scarcely representative for other regions. Here the two-stream approximation appears to be optimal trade-off across the available computational tools. This approximation models the total amount of light returned to the Earth irrespective of geometry and can provide the information on the total amount of skyglow per unit lamp (or per unit ground illumination). Respecting a statistical distribution of ground-based light sources, the average downward radiative flux for a large territory can be simulated easily using two-stream approach. If there is no direct illumination from lamps (like in natural spaces surrounding a town), then the luminance is mainly due to sky glow. This is very often case for small parks or rural regions. In our computations we preferably pay attention to the sky glow effects that are locality independent. The two-stream approach is of great benefit here, since it allows us to retrieve the basic features of visual “perception” of sky glow from different lamp-types. The solution model we used is applicable to compute both the upward and downward radiative fluxes at any altitude. The downward radiative flux comprises the diffuse radiation, while the upward flux is a sum of diffuse and direct uplight.

In principle, the two-stream approximation allows us to determine 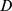 for every atmospheric layer defined during the model configuration. However, only the irradiances at the ground are analyzed in Sec. 3. The input parameters to the computational model comprise: i) lamp emission spectra, ii) spectral attenuation (or scattering) coefficients and vertical stratifications of atmospheric constituents, iii) aerosol characteristics (in particular, the size distribution function, mean refractive index, single scattering albedo, asymmetry parameter), iv) total aerosol optical thickness at a reference wavelength (usually at 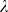 = 500 nm), v) spectral albedo of the earth's surface, and vi) spectral bandwidth. The outputs comprise: i) upward radiative fluxes, ii) downward radiative fluxes. The asymmetry parameter introduced above characterizes the scattering pattern of aerosol particles and can vary from −1 to +1. When being −1, the particles scatter to backward direction. The value of +1 represents a forward scattering. The particles scatter equally to all directions (isotropic scattering) when having the asymmetry parameter equal to 0. However, the asymmetry parameter ranges from approximately 0.6 to 0.9 under real conditions. The single scattering albedo is the ratio of scattering efficiency to total extinction efficiency, so it can vary from 0 to 1. Non-absorbing particles have single scattering albedo equal to 1. If the particles are composed of absorbing materials, then the single scattering albedo approaches 0. We have used the single scattering albedo of 0.95 and asymmetry parameter of 0.75 that are typical values for many regions in Slovakia. The numerical simulations discussed below are made for snow-free surfaces with average albedo of 0.2.

## Results and Discussion

As noticed in Sec. 2, the diffuse illuminance is determined from Eq. (2), where 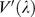 replaces the spectral transmittance function 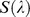. Then, the effects of diffuse radiation on humans can be evaluated in scotopic luxes. The expression scotopic lux is used to explain the illuminance perceived by the human eye under the scotopic visibility function [44], [45]. Unfortunately, the corresponding transformation for insects is scarcely possible due to the unavailability of comparable units (or units that could be used for qualitative and quantitative intercomparisons). For these reasons we decided to interrelate the computational results using the relative perception functions shown in Fig. 1.

The main results of the present work are divided into 5 different topics:

- The flux density in visual spectral range versus sky glow effects that humans can perceive.
- The effect of five light sources in the sky glow perception of living organisms - the lamp systems are configured so that each generates the same luminous flux.
- The effect of fixed number of lamps: unlike the above configuration, the number of lamps will be taken constant regardless for differences in luminous fluxes.
- The effect of aerosol optical thickness (AOT) on the spectral behavior of diffuse radiation as perceived by the three species.
- The effect on visual perception - showing relations between the relative spectra of the lamps and the spectral vision of the three species.

### 1. Irradiance vs. illuminance levels: i.e. photometric vs. radiometric quantities

**Figure 3:**
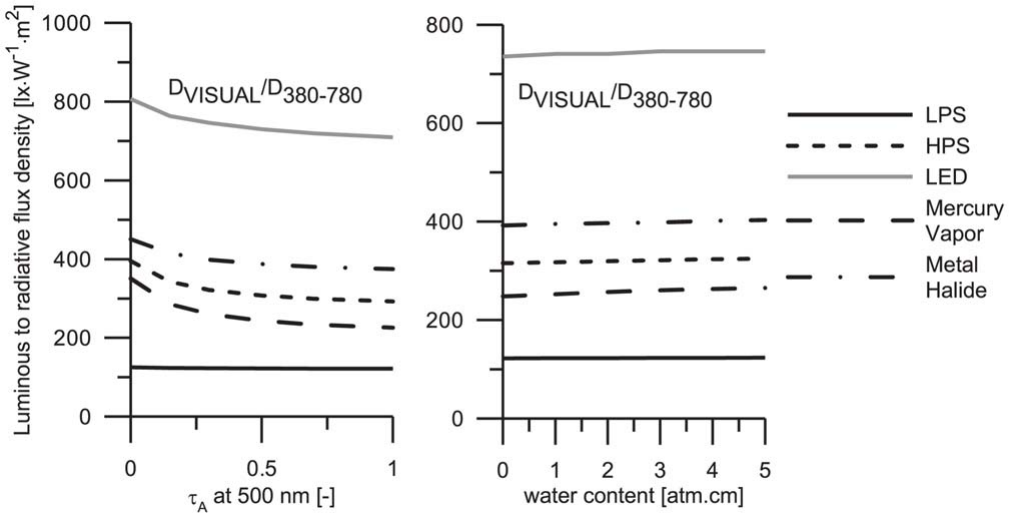
The 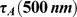 is the aerosol optical thickness at the reference wavelength 500 nm. One of the most interesting features of this figure is the possibility to extract the portions of sky glow levels in VIS spectral band. The aim is to evaluate the radiative fluxes based on the luminous fluxes that humans can feel as sky glow. The evaluated ratios are measured in lx.W^−1^.m^2^.

It appears valuable to determine the ratio among the luminous flux (adjusted to the sensitivity of the human eye) and the radiant flux (the entire amount of skyglow). Such computations can clearly demonstrate the relationship of these two fluxes under evolving aerosol optical thickness or under varying precipitable water content. Interrelating these fluxes one can get a better view on what portion of electromagnetic pollution the humans can perceive. The ratios of luminous fluxes (for scotopic sensitivity of the human eye) and radiative fluxes are shown in Fig. 3. More detailed information on these lamps (considered as potential LP sources) can be found in the Introduction.

In the Fig. 3, the entire amount of sky glow is compared with that perceived by the human eyes. It is demonstrated that the above mentioned ratio is a decreasing function of aerosol optical thickness. However, the situation changes when this ratio is plotted as a function of precipitable water content. In this case, a slight increase of the 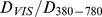 is observed. The main reason for such differences is the scattering phenomena which plays an important role in aerosol optics, but not in water absorption. Note that precipitable water is considered to cause a weak absorption in the VIS spectral range.

### 2. The influence of sky glow in living organisms under constant luminous emissions of ground-based light sources

One of the most significant consequences of LP and sky glow might be the influence to living organisms. In this section we analyze the effects that diffuse light can cause to the species studied. The choice of constant luminous emission is advantageous since it allows us to compare the spectral features of sky glow produced by different lamps independent of lamp power. In the Tab. 1 we considered the lamps that are typically installed in public spaces across different countries and regions. These lamps differ in their powers. For instance LPS lamp operates at 18 W, while HPS lamp has 170 W. Normally it would be difficult to compare sky glow levels generated by these light sources. However, the normalization regarding luminous emission makes such a comparison possible. In this normalization we adjusted the number of lamps so that their bulk luminous powers become equal and thus undistinguishable by human eyes. As discussed above, four LED lamps are necessary to produce the luminous energy equivalent to five HPS lamps. In another example, five MH lamps may mimic the luminous emissions produced by two HPS lamps. Having this base fixed, a reasonable comparison of sky glow levels for these lamps can be made in different spectral bands. Without the previous mentioned normalization, the effects of different lamp types on sky glow cannot be interrelated correctly. Since this concept has the theoretical foundation it can be used for any lamp-type.

**Figure 4:**
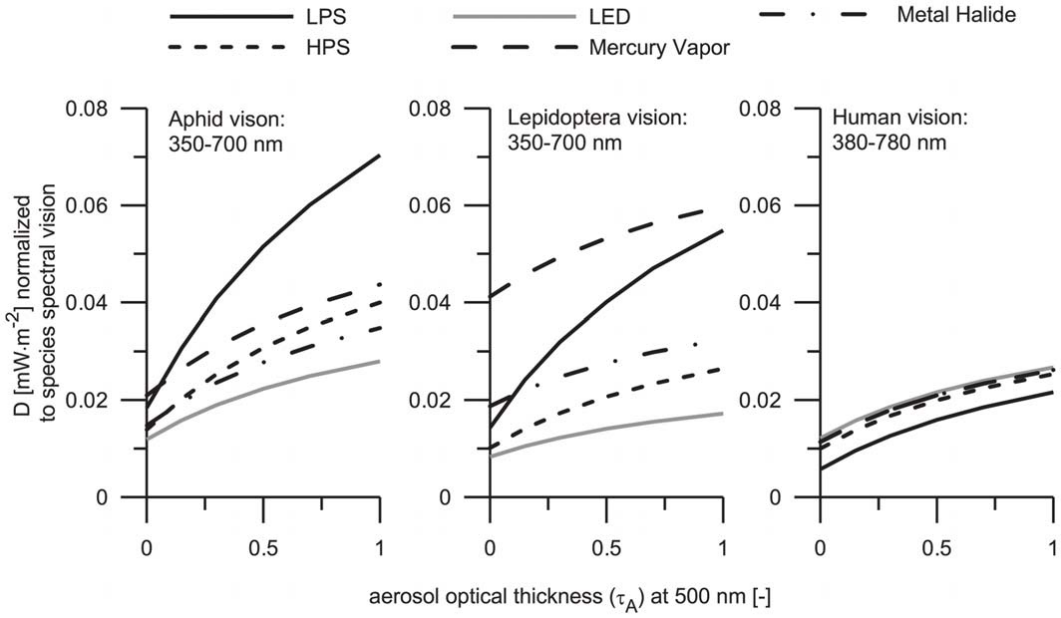
Total amount of precipitable water is 0.3 atmospheric centimeters. The curve styles coincide with those that have been used in Fig. 3. The results were obtained under assumption of constant luminous emission to the upper hemisphere; the computed flux densities are normalized to the visual sensitivity of the organisms selected for the present study.

**Figure 5:**
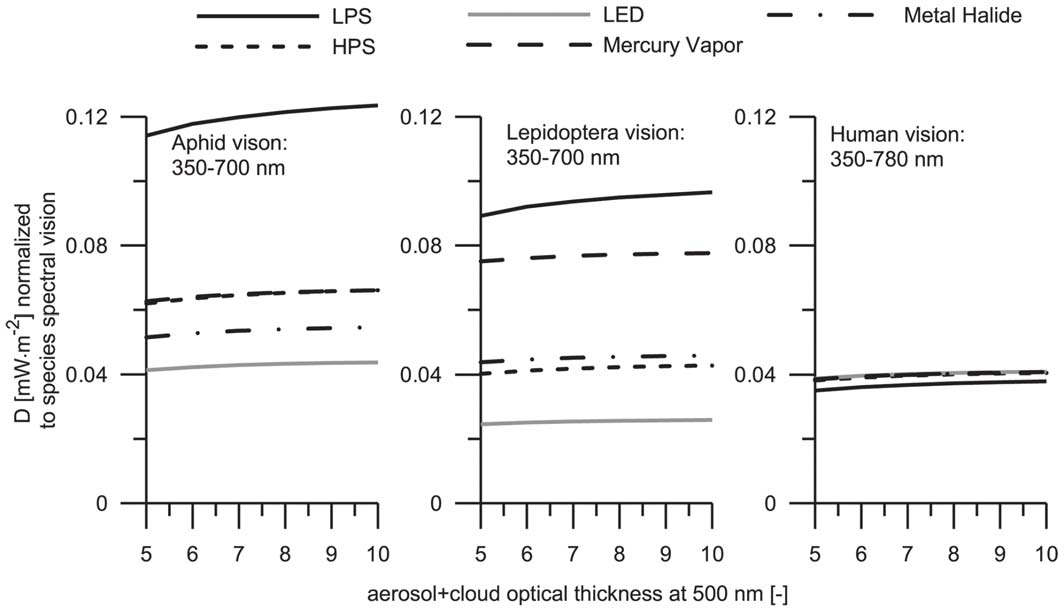
The water content is the same as in **Fig. 4**. To identify the curves see the legend in Fig. 3. The results were obtained under the same assumptions as in Fig. 4.

The flux densities computed under clear-sky and overcast-sky conditions are depicted in Figs. 4 and 5, respectively. A growth of sky glow levels at elevated aerosol optical thickness is apparent for all light sources. It is because the extinction coefficient in a slightly turbid atmosphere is small enough to cause any significant attenuation of the scattered radiation. In other words, the scattering efficiency is a dominant factor influencing the diffuse irradiance under low aerosol optical thickness conditions. An average value of 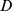 for the overcast sky is approximately four times higher than in for clear sky conditions. Such an effect is related to the diffuse reflection by a cloud base.

One of the important features emerging of these findings is the determination of sky glow perceived by the species. Assuming that the public lighting system is designed to generate a constant luminous flux, independent of lamp-type, the alate m. persicae night vision is most sensitive to sky glow from LPS lamps. It is important to emphasize this result since LPS lamps are considered to be insignificant pollutant sources. Our finding can be demonstrated clearly by analyzing the spectral sensitivity of the alate m. persicae (Fig. 1), which peaks at λ≈527 nm. This aphid shows enhanced sensitivity to wavelengths 450–600 nm, while the spectral radiance of LPS lamp is located between 580 and 600 nm. Considering the spectra of other light sources it is apparent that sky glow from MH lamp and MV lamp show similar effects, while sky glow from HPS lamp and LED lamp are causing the less effect to the visual perception of this insect. The aphid is a phytophagous pest of field crops and greenhouses, having secondarily the consequence of several virus diseases. It is suggested that some kind of yellow light could attract this insect, thus resulting in an easy localization of plant targets [46]. Note that LPS lamp emits mainly yellow light. Therefore, one can assume that if this type of sky glow is found near the field crops and greenhouses, then it might enhance the possibilities of having agricultural pest problems. The effect of sky glow has not been previously studied and is correspondingly necessary to have the adequate natural conditions to the propagation of the pest (like the temperature, humidity, among others).

The narathura bazalus is more sensitive to sky glow from MV lamp under clear sky conditions. Note that this type of lamp has significant radiance in the UV spectrum (see Fig. 2) and the lepidoptera has a high sensitivity to this spectral band showing a primary peak at 340 nm. The effects of sky glow from LPS lamp are similar to those produced by MV lamp when the reference aerosol optical thickness is as high as 1. However, the situation changes under overcast conditions; then the butterfly is more sensitive to sky glow from LPS lamp. Under both clear and overcast sky conditions, sky glow from LED lamps only slightly influence this lepidoptera. Previous studies have reported that LED lamps cause not attraction to moths (insect of the order Lepidoptera) [47].

No significant differences to the human vision were found since the emissions by different light sources are normalized to the same luminous fluxes. Any small alterations of sky glow levels are related to the optical properties of undercloud atmosphere. Specifically, the humans are most sensitive to sky glow from LED lamps closely followed for that produced by MH lamps and MV lamps. Finally, we can conclude that apparently LPS lamps and HPS lamps have only minor effects on sky glow perceived by humans.

### 3. The influence of sky glow in living organisms assuming constant number of light sources

**Figure 6:**
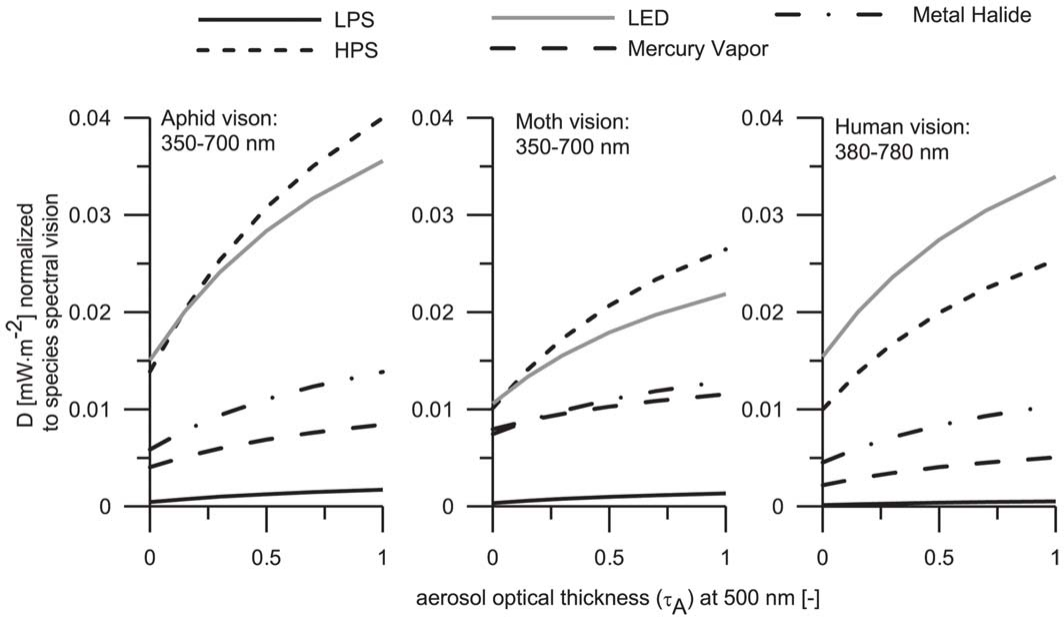
Total amount of precipitable water is 0.3 atmospheric centimeters. The curve styles are the same as in Fig. 3. The results were obtained under assumption of constant number of light sources and the computed flux densities are normalized to the visual sensitivity of the organisms selected for the present study.

**Figure 7:**
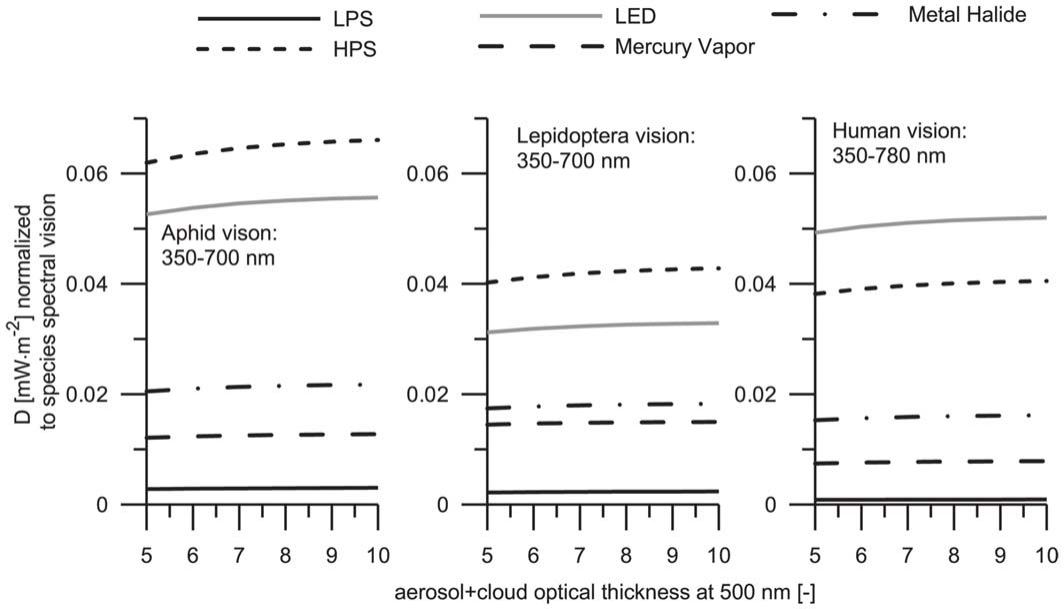
Total amount of precipitable water is 0.3 atmospheric centimeters. The legend to the plots is the same as in Fig. 3. The results were obtained under the same assumption as in Fig. 6.

A conventional situation with constant number of luminaries is analyzed below. Similarly as before, sky glow is normalized to species spectral vision with the aim to determine the effects that light sources would have on the organisms and analyzing the situation when one lamp type is replaced by another independent of Wattage. This analysis simulates the condition in which a constant illuminance is considered independent of a type of light source used. The computational results are summarized in Figs. 6 and 7.

In contrast to earlier findings (Sec. 3.2 of this paper), the Figs. 6–7 demonstrate that the insects are more sensitive to sky glow from HPS and, with similar characteristics, LED lamps. Although the spectral sensitivity of alate m. persicae is similar to that of humans (see Fig. 1), both these species would perceive sky glow in a different way. Most typically, such discrepancies between sky glow levels are observed under clear sky conditions when the aerosol contamination is rising. On one hand, humans perceive sky glow emitted by HPS lamp less efficiently than alate m. persicae. It is due to enhanced sensitivity of the aphid to the UV spectrum and then at wavelengths 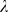
>500 nm. On the other hand, sky glow from LED lamps influence human likewise aphid. Since the spectrum of LED lamp is characterized by a wide continuum, humans and alate m. persicae have nearly the same response and thus no dramatic differences are found between left and right panes in Fig. 6 (see gray curves).

Analyzing narathura bazalus and human visions, it can be seen that sky glow from HPS lamp cause nearly the same effects to both. However, the humans are most sensitive to sky glow from LED lamp. This is of course related to the similarities between the human spectral sensitivity and the emission spectra of LED lamp (human scotopic sensitivity ranges from 400 to approximately 620 nm, while the main radiance of LED lamp is located between 400 and 650 nm with two peaks in 450 and 550 nm). Previous studies have reported that the vision of lepidopterans is mainly sensitive to short-wavelength lamps and that LED lamps cause less attraction to them e.g. [47]. Our results obtained under assumption of constant number of lamps suggest that narathura bazalus is more sensitive to sky glow from LED lamps than to other lamps conformed mainly by short wavelengths. The final consequence of LP to moths is the biodiversity crisis [23]. sky glow attraction has been not previously studied considering this specie, but our results indicate the different perceptions. Previous studies have reported the effect of sky glow in other insect species [1], and it can be assumed that the effect in lepidopterans may be similar.

Concluding this section, we can see that sky glow from LPS lamp shows the minor effect to all three species. This finding is consistent with the spectral radiance of LPS lamp that shows only a narrow peak between 580 and 600 nm.

### 4. The effect of AOT in the diffuse radiation

**Figure 8:**
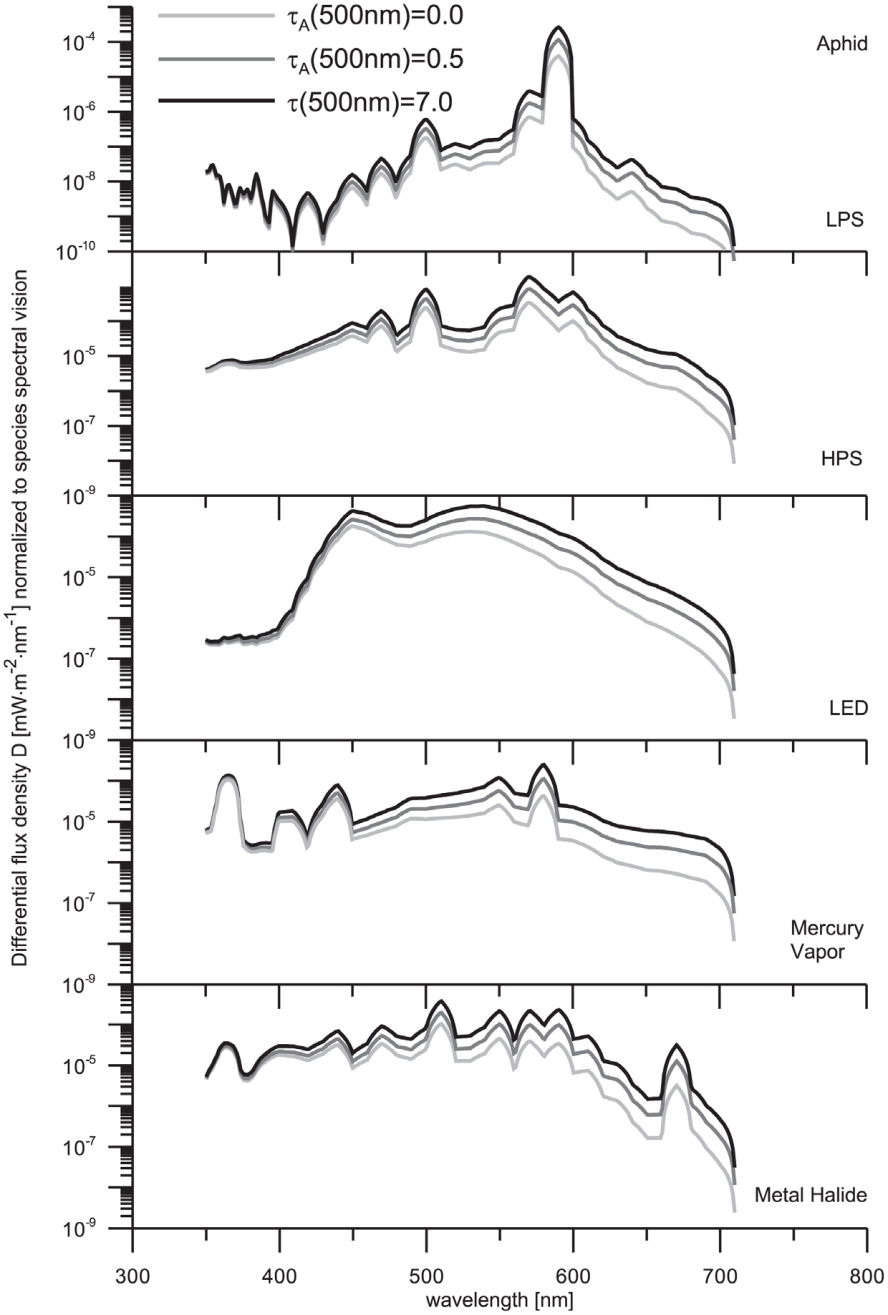
Total amount of precipitable water is 0.3 atmospheric centimeters. Two models are used to characterize clear sky conditions: no aerosol contamination (light-gray curves) and aerosol contamination characterized by optical thickness 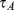 = 0.5 at 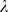 = 500 nm (dark-gray curves). Consequently, the spectral features of sky glow under overcast sky conditions with total atmospheric optical thickness 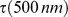 = 7.0 are correspondingly determined (black curves).

**Figure 9:**
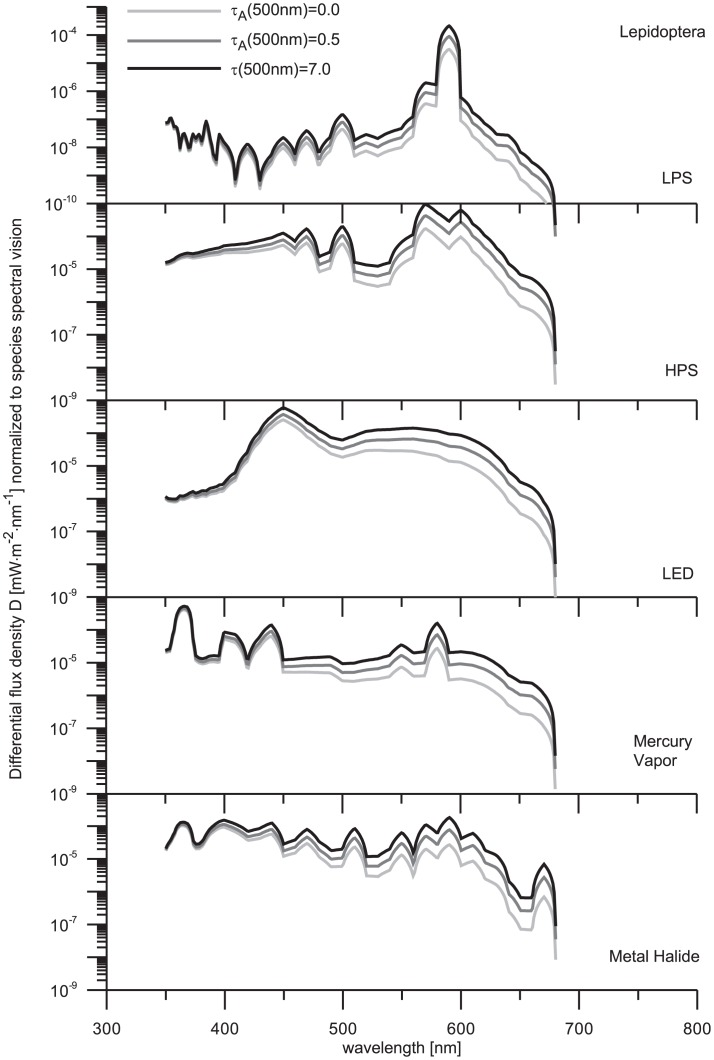
The legend to this figure is the same as in **Fig. 8**.

**Figure 10:**
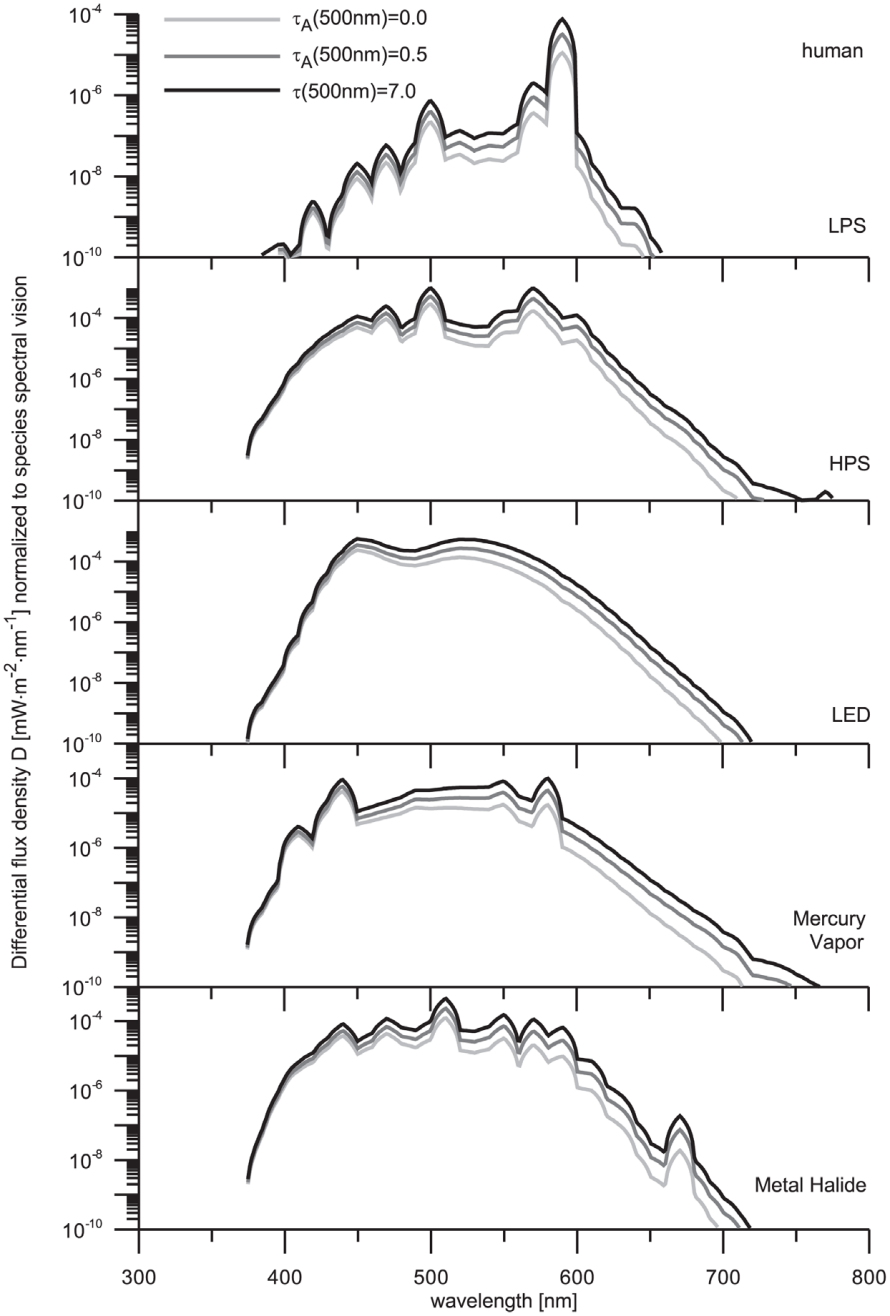
The legend to this figure is the same as in **Fig. 8**.

The overall effects of sky glow on species vision have been demonstrated in the earlier chapters. The differences found are generally related to the specific spectral features of both light sources and species perceptions. To recover some of these features we also computed the spectral profile of 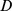 that is derivative according to the wavelength. The numerical results are depicted in Figs. 8–10, where the physical quantities plotted have dimensions W⋅m^−2^⋅nm^−1^.

The growth rate of differential flux density (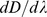) shows the same trend for all lamps. However, some effects are artificially suppressed as a result of the logarithm scale used at y-axis in all figures. Despite of this effect, we have preferred log-scale graphics since the linear scale is not convenient to display 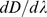 varying over several orders of magnitude. Commonly, the 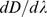 tends to be independent of atmospheric turbidity at UV edge of the VIS spectrum (i.e. there are not many differences between the three optical thickness conditions). A comparison with the VIS spectrum reveals that these differences are growing with the increase of wavelength. That result is related to the elevated UV optical thickness even if the aerosol content is low. Namely, the Rayleigh optical depth at 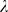 = 350 nm is fairly high (more than 0.6). In this case, the volume attenuation coefficient of the atmosphere is large enough to guarantee an efficient removal of electromagnetic energy. Even if no aerosol contamination is considered, the intensity of a beam propagated upward and scattered toward the ground is reduced by a factor (e^−0.6^)×(e^−0.6^)≈0.3. This value is further reduced because of low backscatter efficiency. The effect of aerosols is more evident at larger wavelengths, where 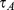 significantly exceeds the Rayleigh-component 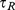. For instance, 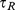 = 0.36 at 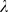 = 400 nm, while 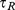 = 0.036 at 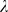 = 700 nm. Let us now assume the turbid atmosphere characterized by 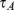 = 0.5 at the reference wavelength 500 nm. A set of experiments made in urban and suburban regions has shown that 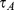 often behaves like 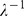 (e.g. [49], [50]). In this case, we obtain 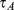 = 0.63 at 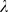 = 400 nm and 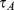 = 0.36 at 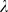 = 700 nm. One can immediately recognize that the ratio of 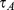/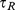 is smaller than 2 at blue edge of the VIS spectrum, but it grows up to 10 at the red edge of the VIS. Therefore, the effect of varying aerosol optical thickness dominates just at large wavelengths (see Figs. 8–10).

In the current findings, we have shown that alate m. persicae is sensitive to the changes of atmospheric and optical conditions. It is evident that alate m. persicae have a strong response to the visual range of the spectrum and also have specific wavelength dependence. An increase of sky glow with growing aerosol optical thickness is well identifiable for all five light sources in the VIS spectral range. Due to a bit enhanced sensitivity of the aphid to UV radiation, this insect can be sensitive to sky glow from MV lamp emissions. Contrary to the traditional expectations, sky glow from MV lamp influences the alate m. persicae significantly in the VIS spectral band. The narathura bazalus is predominately sensitive to sky glow from UV spectrum, so the effect of light sources is more apparent between 350 and 400 nm with the exception of both sodium vapor lamps. It reflects the fact that these discharge lamps have not important emissions in UV part of the electromagnetic spectrum. These results further support the idea that this lepidoptera may have a reaction to the atmospheric turbidity conditions.

### 5. Effect of species visual perceptions

In order to understand the effect of the five lamps on the visual perception of the species, the relative spectra of the lamps were considered with the purpose to perform normalization to the spectral sensitivity of the three species. This normalization allows us to identify the direct lamp emissions that the specie can perceive. It can be seen that the narathura bazalus is rather sensitive to sky glow from UV part of spectrum and this may explain most of the optical effects that light sources operating in spectral range 350–400 have in this insect. These findings are consistent with the idea of that MV lamp could have important UV effect on this insect. As documented in Figs. 6 and 7 the narathura bazalus is sensitive to sky glow from both HPS and LED lamps. This is due to the fact that HPS and LED lamp emissions are observed across a wide range of VIS spectrum (between 450–600 nm) and lepidoptera vision is limited to the spectral band 420–600 nm. LED lamp has two important radiance peaks; the main peak is located between 400 and 500 nm and a smaller but more encompassing peak is located between 500 and 700 nm. The first peak is perceived in a greater extent by the lepidoptera. Also the UV part of HPS lamp is highly related to the spectral sensitivity of narathura bazalus. This type of lights could enhance the flight-to-light behavior [48] and attraction [21], which are the most common effects that light sources produce in lepidopterans, mainly moths. A possible explanation for this phenomenon might be that some lepidopteran species are guided by the moon. They mistake strong lamps and fly to them crashing at high speed, which kills them [48]. The attraction of lamps to lepidopterans increases the predation by birds, spiders and mainly bats; probably that is the reason why several groups of bats are surrounding the street lamps.

**Figure 11:**
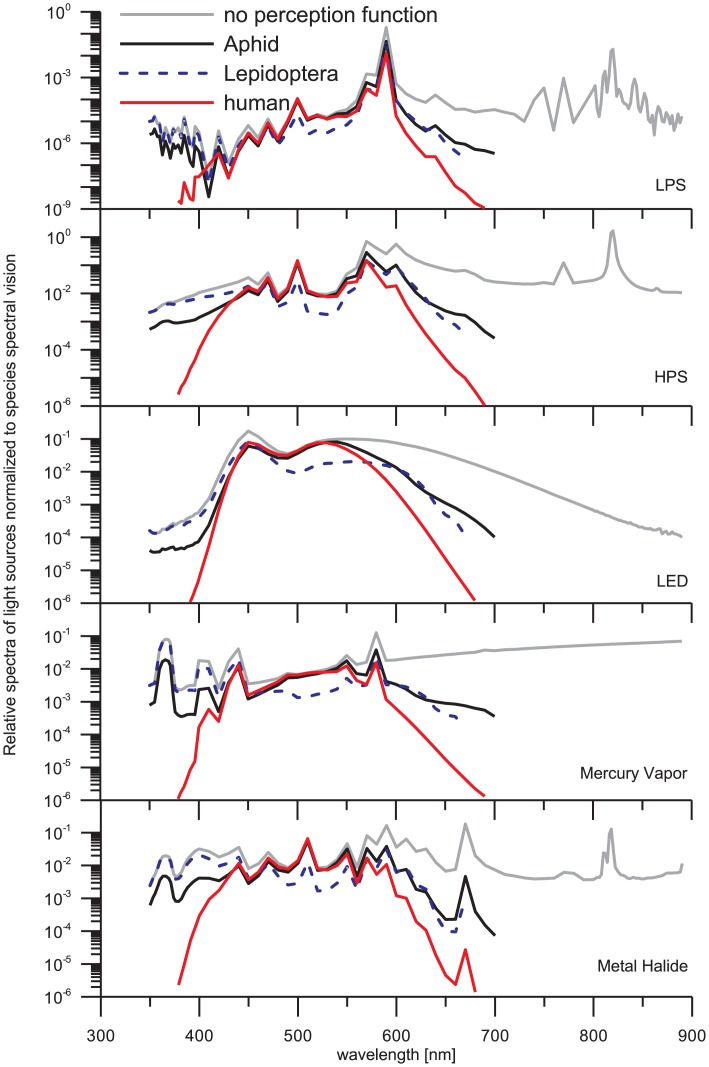
A comparison contrasting the relative spectra of the lamps and the spectral vision of the three species is shown in this figure. The main objective is to see clearly the differences between the lamp emissions and to relate them to what the species can feel.

The sensitivity of the human eye is more or less similar to the alate m. persicae in the VIS spectrum. Unexpectedly, HPS lamp is causing more effect to alate m. persicae while LED lamp influence mostly humans. The reason for such behavior is the relatively important UV sensitivity of aphid. LPS lamp emits in a very narrow spectral band, typically between 580 and 600 nm and some negligible quantities in UV spectrum. Nevertheless, LPS lamp is the less pollutant source for all three species, including humans. As it can be observed in the Fig. 11 some light sources have important emissions in the IR spectrum, but the effect on species vision is insignificant.

## Conclusions

A large fraction of artificial night lighting comes from public light sources. To recover their effects on sky glow levels it is necessary to analyze different installations of single lamps in the urban environment. The distribution of electromagnetic energy in the lamp spectra plays a crucial role in understanding the problem, as well as the intensities of the emitted radiance.

In the present study, the characteristics of sky glow were analyzed under different conditions and for different parts of the electromagnetic spectrum. Comparisons were made examining the sensitivity of three different living organisms including humans. The current investigation was limited to the human scotopic vision since the sky glow and emissions were evaluated against a common base (as explained in Sec. 2). It has to be stated that new set of computations for intermediate (mesopic) vision and also for photopic vision are designed, but the results will be presented in future studies. Based on the results and discussions given in the previous chapters, the next two conclusions can be made:

- LPS lamp could be the best to avoid sky glow that generally makes the astronomical observations difficult. Namely, the astronomers can use a special filter to eliminate narrow-band emissions of LPS. However, sky glow from LPS is the worst to the vision of the insects tested when a constant illuminance of urban spaces has to be guaranteed independent of the type of light source used. Regarding the public lighting, LED lamp would be the best light source to avoid sky glow effects in these particular insects. That is the reason for inconvenience of LPS lamp if a population nears the natural spaces requires a certain (fixed) amount of luxes. Under the same conditions, the human eye is more sensitive to sky glow from LED lamp and in smaller extent to LPS lamp. The main influence of artificial light in the aphid is the attraction [17], [51], [21]. This situation could facilitate the localization of field crops and greenhouses, promoting the developing of agricultural pests and the imbalance of the normal plant grow rates. It has been reported that aphid is more sensitive to the yellow light [46]. Therefore one can assume that LP and sky glow from LPS lamp could increase the propagation of aphid pest, when a constant illuminance at the ground is required.
- The attraction represents also the main LP problem to narathura bazalus, nevertheless in a different way than alate m. persicae. This butterfly could have tendency to flight toward the light depending on the type of light source. Having the fixed number of luminaires, sky glow from HPS lamp causes more effect on the vision of insects closely followed by sky glow from LED lamp. This might imply possibly a greater attraction with HPS lamp, but this differs from previous observations in other lepidopteran species [16], [17], [47]. The human vision is sensitive to sky glow from LED lamp closely followed by sky glow from HPS lamp. Nevertheless, sky glow from HPS lamp causes an effect in the same quantities for humans and narathura bazalus. From sky glow point of view, the LPS lamp we choose for this study would be the best choice for all three species, when is not necessary to guaranteed a constant illuminance at the ground.
